# Gynecology

**Published:** 1876-04

**Authors:** 


### VII. GYNECOLOGY.

1. *Treatment of Chronic Corporeal Endo-Metritis by Pressure.* PROF. T. A. REAMY, of the Med. Col. of Ohio. (*The Clinic.*)

The Professor advocates the use of laminaria tents, carried to the fundus uteri. The cavity of the uterus is filled full of tents and they are allowed to remain till uniform pressure has been exerted upon the corrugated, reduplicated folds of the sodden endo-metrium, ironing as it were, (or *sponging*) them out as smoothly as can be accomplished by this pressure from within outwards. First, one long tent is inserted extending from the os uteri externum to the fundus; then shorter ones are introduced reaching from the os *internum* to the fundus, numbering from two to four, according to the capacity of the body. The shorter ones surround the long one. The effect of this pressure upon the lining membrane of this organ is to cause it to respond much more readily to remedies, which can be much more easily applied. Six cases has the learned Professor treated thus, and he calls the attention of the members of the Cincinnati Academy of Medicine to this style of treatment by pressure.

(This subject is treated of by Prof. Byford only, in his work on the uterus. The *alterative effect* of pressure is there dilated upon. Professor Reamy is referred to that work.—ED.)

2. *Fibroid Tumor of the Perinæum.* DR. ELY McLELLAN, U.S.A. (*Louisville Med. News.*)

The patient, Miss A., æt. 23 years, a seamstress by occupation, suffered greatly from the presence of a tumor occupying the perinæum, encroaching rather more on the vagina than the rectum, and inducing symptoms almost identical with those of hæmorrhoids. Its growth had been slow and was first noticed after wearing a disk pessary a few days. The patient, thoroughly anæsthetized, placed in the BREECH BACK position, was operated on as follows: The vaginal covering of the tumor was placed upon the stretch by means of retractors in the vagina and the operator's forefinger in the rectum pressing behind the tumor. An incision to the extent of the tumor along the vaginal raphé was made, and the vaginal portion carefully dissected off. Failing to remove the tumor by traction with the vulsellum, the mass was removed by the knife, in accomplishing which, the finger in the rectum was of invaluable service; for so close to the rectal wall was it necessary to go that the anal circular fibres were distinctly demonstrated. Recovery speedily took place. The tumor, of the size of a hickory nut, possessed no distinct envelope, "differed only in density and color from the surrounding tissues, was distinctly an outgrowth from the sphincter ani, and its cause was undoubtedly to be found in the local irritation induced by the use of the pessary." The extreme rarity of the location, and the undoubted cause (presenting an additional objection to such pessaries) of this tumor, are the reasons for writing its description.

3. *Uterine Hiccough.* FRITSCH. (*Medical Times; Med. and Surg. Rep.*, March, 1876.)

The patient, a young married woman, previously in good health, had been suffering from hiccough three months. No cause could be assigned. The paroxysms would come on at irregular intervals every day and disturb her more or less during the night. The menstruation was not disordered, but with it the trouble exacerbated. A great variety of therapeutics heretofore resorted to, had failed to give permanent relief and the patient's health was now evidently suffering. Vaginal examination revealed excoriations at the portio vaginalis uteri, with moderate cervical catarrh. In every other respect, the reproductive organs were found healthy. Local treatment with the solid silver nitrate was followed by a prompt arrest of the hiccough and ultimately in a complete cure of the primary disease. This case strikingly illustrates reflex manifestation.

